# Supplementary material for: Effects of COVID-19 lockdown on heart rate variability
Source: PLoS One. 2020 Nov 12;15(11):e0242303. doi: 10.1371/journal.pone.0242303 (PMC7660489; doi:10.1371/journal.pone.0242303)
Supplement: Attachment — Submitted filename: REV PLoS - 3.docx [file pone.0242303.s003.docx]

**Reviewer #2: Firstly, I would like to congratulate the authors by their effort in providing clarifications and incorporating adjustments throughout the manuscript.
Most of the issues previously pointed were solved, although some of them were not fully clarified. Considering that, I tried to be more specific in some requests and reinforced the importance of them for the higher quality possible of this manuscript.
Some minor aspects not highlighted in the previous review were pointed now, but mostly as suggestions for a better readability of the manuscript.
Methods section still brings me some concern, but I hope that with authors adjustments and clarifications we will turn that section even better.**

We thank you for reviewing our manuscript and for providing comments and suggestions that have helped us to improve it. We have considered your remarks and made amendments when necessary in the revised manuscript. We appreciate your further perusal of the revised manuscript.

We have provided our responses to your comments that are in **bold**. Amended sentences are in italic with the additional wordings in red.

**Dear author,
Find below my review of your revised manuscript, divided by section:

Abstract
The adjustments made in the abstract were well done, congratulations.
However, I added some points that still needs attention, that follows:

I suppose that adjusting the following sentence to the plural would be appropriate:
“there was a moderate significant correlation between VAS and HR, RMSSD, HF, respectively, in the supine position”.**

Done

**Also, I noted that is a significant correlation between standing RMSSD and VAS present in table 1. Comments about that result are missing in the abstract and throughout the manuscript.**

Thank you for this relevant comment. We modified the sentence as follows:

*In standing position, HRV parameters were not modified during CFN but RMSSD was correlated to VAS.*

**Finally, I also suggest you to suppress the term “moderate” of the above affirmation or provide, for the readers (in the manuscript body), the parameters you adopted for that qualitative interpretation of the Pearson’s correlation coefficient.**

Done **Regarding the abstract’s conclusion I would suggest you rethink the ending of the final sentence, especially considering that, although broad, your sample may not represent the general population. In my opinion, simply changing “in the general population” to “in this population” may solve this issue.**

Done

**Introduction
PARAGRAPH 1:
The adjustments made in the first paragraph are well done.**

Thank you!

**PARAGRAPH 2:
Regarding the second paragraph, the quotations on the first sentence (references 6 and 7) remains not supporting the affirmations as they are. However, you may solve that issue simply, including “some of the possible” after: “…but also in mitigating”; and adding “ In those cases” before: “Aerobic exercises such as walking or endurance…”. Or another way, feel free.**

We followed your relevant suggestions. The sentences are now:

*Exercise plays a fundamental role in cardiovascular health [5] but also in mitigating some of the possible psychological impacts of lockdown [6]. In those cases, aerobic exercises such as walking or endurance running appears to be more beneficial than sprint running or force/power exercises [6,7].*

**The second paragraph part referring to HRV indexes was really improved.
But the sentences referring to the LF HRV index still needs a little adjustment. Since band migration of the RSA is not the only issue regarding the physiological meaning of LF, I would suggest you complement the text alerting the readers about that.**

*The migration of the respiratory sinus arrhythmia in the low-frequency band is a subject of debate [15], and it is not the only issue regarding the physiological meaning of LF [16], which leaves the physiological implications of this frequency band unclear [17].*

**Also, please complement the quotation of the sentence that refers to HF and RMSSD, Reference 18 only supports the HF index physiological meaning, but not RMSSD index meaning.**

We have added the reference to the task force (1996) to help the readers to understand the physiological meaning of RMSSD.

**Also, please, better clarify for the readers what are you trying to say with “of one or the other” in the following sentence: “Hypotonia of one or the other is a sign of cardiovascular deconditioning”. Or you may suppress “of one or another”, as the sentence “Hypotonia is a sign of cardiovascular deconditioning” sustain the idea of the previous sentence and reads better, in my opinion.**

We modified the sentence as suggested. **PARAGRAPH 3:
In the third paragraph the hypothesis adjustment was all right. However, the study still lacks an explicit justification, and the second aim is a little over tuned. But I think that there is no need to jeopardize the publication by focusing on this points more related to my perception. So, I will let those observations for your reflexion.**

You are right. We toned down the sentence as follows:

*..and to investigate whether changes in HRV were related to some changes in psychological states during and after this period.* **Method
Most of the adjustments made in Methods section are all right. However, some aspects still brings me concern.

Participants:
Thank you for the explanation about the age range of your sample.
To explicitly declare some information like that, in this section of the manuscript, can improve the comprehension of methodological processes that may not be obvious for the reader. So, please, consider to include something like the sentence that you wrote in the response letter: “… The initially planned studies involved physical exercises with a maximum age for inclusion criteria of 60 years-old to limit the cardiovascular risks”; or another similar statement. That would be fine.**

Thank you again for this excellent suggestion. We added this sentence in the manuscript.

*The initially planned study involved physical exercises with a maximum age for inclusion criteria of 60 years-old to limit the cardiovascular risks.*

**Still regarding inclusion criteria, a request was not fully clarified. So, I will try to be more specific: You state that “physically active, healthy, non-smokers, with no known diseases and no pregnancy or lactation for women” subjects composed the sample. If all those data were self-declared, please, make a statement on the manuscript clarifying it. Or, if, for example, physical activity or health were measured by accelerometry or (previous to the pandemic state) physical examination, respectively, you may report which data was declared and which was measured, and how it was done.**

We modified the sentence as follows:

*All subjects self-declared being physically active, healthy, non-smokers, with no known diseases and no pregnancy or lactation for women as well as living in France.*

**Thank you for the explanation regarding the adoption of forty tests instead of thirty. Please, consider incorporating on the manuscript body part of the explanation provided in the response letter. Explicitly declaring some methodological decisions like those could reinforce the quality of your work, for the readers.**

You are right. It would improve the quality of this work. The following sentences were added:

*More specifically, from January 1^st^ to May 11^th^ (end of lockdown) there were 20 weeks. With 2 tests per week in average per participant, 40 tests were performed.*

**Heart rate variability:
Thank you for the information about the HRV measurement devices.
Please, make a quoted statement on the manuscript body about the validity of the adopted HRV measurement devices. You may simply adjust part of the following explanation provided in the response letter and concisely incorporate it to the manuscript:
“The H7 is the most used RR-recording device, the TP5+ uses the exact same technology:
Comparison of Heart-Rate-Variability Recording With Smartphone
Photoplethysmography, Polar H7 Chest Strap, and Electrocardiography. Daniel J
Plews, Ben Scott, Marco Altini, Matt Wood, Andrew E Kilding, Paul B Laursen. Int J
Sports Physiol Perform. 2017 Nov 1;12(10):1324-1328. doi: 10.1123/ijspp.2016-
0668. DOI: 10.1123/ijspp.2016-0668.**

**The smartphone application is just a Bluetooth receiver, using the standard BLE
protocol, which does not interfere in any ways with the recording. It is just a wireless
system. The results would have been exactly the same if the RR intervals had been
stored on the smartphone memory and downloaded to a computer using a wire.**
[**https://web.archive.org/web/20170130085524/https://www.bluetooth.com/specificatio**](https://web.archive.org/web/20170130085524/https:/www.bluetooth.com/specificatio) **ns/bluetooth-core-specification”**

We followed your recommendation and have incorporated this information in the manuscript as follows:

*The H7 is the most used RR-recording device, the TP5+ uses the exact same technology [23]. The smartphone application is a Bluetooth receiver, using the standard BLE protocol, which does not interfere in any ways with the recording.*

**Regarding the artifact corrections, please, state in the manuscript body that your app uses filtering algorithms not available to the public that were applied in that process, since that information may impact the replicability of your data.**

We added this information as follows:

*The RR intervals from the orthostatic tests were first analyzed to remove ectopic beats from the recordings, the algorithms used are currently not available to the public.*

**Visual analogue scale:
Thank you for the complement to the manuscript about the remote support provided to the subjects. I also agree that VAS is a widely used tool. However, the way that your VAS was presented in the manuscript lacks some important information. I’m afraid that “their sensation of the moment”, as you state in some point, is a poor description and may have several meanings beyond the intended in the present study.
An adequate presentation of that psychometric instrument needs a more detailed description in the methods to ensure an adequate replicability of your study. So, I would like to suggest you report in this subsection of the methods some aspects as the anchor question, and the values on the horizontal line extremes, that are missing. Please, provide and incorporate to the manuscript a more detailed description of that psychometric instrument (VAS) that you adopted, since as states the article provided by the authors in the response letter: “The most important aspect of any VAS however is the question that is combined to it and not the line. The line remains the same, but the question may change.”.**

We understand your concern but the question was on purpose very vague as we aimed to obtain a general feeling of the subject. We did not focus on a special component of the mood as vigor, anxiety or anger. Here we display a screenshot (in French) of the VAS to clarify this point.

Since it is a limitation of the present study, we added the following sentence in the manuscript:

*Finally, the VAS allowed to only rate a “general well-being” but not any specific mood components as vigor, anxiety, anger etc. Therefore, it remains vague and incorporates probably different psychological states among participants.*

**Correlations:
Thank you for the inclusion of the information in this subtopic. That subtopic should be part of the Statistics subtopic, on my opinion. Also, please, clarify if you tested LF and HF in absolute units or normalized units, or both. If both were tested, you need to include the results of both in table 1.**

As suggested, we moved this part to the “statistics” chapter.

We tested LF and HF only in absolute units. Since LF and HF are labelled nLF and nHF, we don’t believe that we should clarify in the manuscript.

**Statistics Section
Thank you for the explanation regarding the choice of a change in VAS>2 as a criterion adopted for the group division. Concisely reporting that information in the manuscript body may improve the presentation of your method section for the reader.**

Again, your relevant comment helps us to improve the manuscript. The following sentence was added:

*Regarding the group effect division adopted (WB- vs WB+), the criteria was a change in VAS >2, that corresponded to 20% change in the scale. Such a large change aimed to lower the intra-individual variability.*

**Adjustments made in the manuscript regarding the other possible group divisions are well done.

Regarding the statistical power, in addition to presenting values you must declare in the statistical section which way you took to find those values. Please, clarify for the reader how those statistical power values were calculated.
I would like to add that the requests regarding statistical power, in that and in the previous review, were made to add robustness to your method, since the amount of data collected seems to ensure a high statistical power. So, adequately presenting that information in the methods (for both tests: ANOVA and Pearson’s correlation) could highlight a valuable aspect of your manuscript.**

The statistical powers were calculated using the F-statistic values and the degrees of freedom, according to Scheffé, H. (1959), The Analysis of Variance. New York: John-Wiley. p. 227 ; and Zar, J.H. (1999), Biostatistical Analysis (2nd ed.). NJ: Prentice-Hall, Englewood Cliffs. pp. 195-196. Those references were added in the manuscript as well as a short explanation about the computations.

*Statistical power SP is reported for each ANOVA performed, the computations take into account the F-statistic values (expected and observed) and the degrees of freedom [25,26].* **Results
Although partially complete, the previous requests regarding results section were well done.
Please, present in the manuscript the HR and HRV mean values (and SD) of the sample, in each recording position per period (BLS, CFN, and RCV) and per group in each period.**

For not being redundant with the figures 1 and 2, where the means (and SEM) are displayed, we politely disagree to report once more the values.

**I also noted that a significant correlation between RMSSD and VAS was present in standing position (table 1), but the manuscript only mentions and discuss the associations observed in supine position. Please, adjust that throughout the text.**

We added this information in the abstract and in the manuscript, as follows:

*Moreover, RMSSD in standing position was correlated to VAS.*

**Thank you for your consideration about the interactions between HR and HRV. In my opinion, just reporting the mean values of HR and HRV in each position and per period could provide sufficient information for the reader.**

Please see our reply above re. the redundancy to report these values in figures and in manuscript. **Please, clarify which of the reported results of LF and HF are referent to absolute units, and which are referent to normalized units, as you mention that both were calculated in the methods section. In case of using only one of those, I suggest you present in methods section only what was analyzed and reported in results section.**

**The changes in parameters were analyzed using normalized values while the correlations were computed using absolute values. Sentences have been added in the Result section**

All these changes are reported in normalized units.

All correlations were computed on absolute values. **Discussion

Thank you for considering the previous suggestions and for incorporate them in your discussion. However, I would now suggest you some minor and more specific adjustments in your discussion since I have made just a shallow approach to that part of the manuscript in my previous review:

At the ending of the second paragraph of the discussion I suggest you to be cautious with some affirmations. With that in mind, please, adjust the following sentence: “In this increasing well-being, the fact that increased RMSSD, LF and HF (associated to decreased HR) were also observed post lockdown, is a marker of an effective and lasting improvement in cardiovascular fitness.” I suggest you change “is” to “may be” before “a marker” and to change “cardiovascular fitness” to “cardiovascular health”.**

Thank you. Done.

**Also, in the subsequent sentence, I suggest you adopt other expression instead of “performance”, it may sound out of context, since the paragraph is not talking about entrainment or performance specifically, but in part about physical activity, that is a broader concept.**

You are right. We modified “*performance*” for “*responses*”.
 **In the beginning of the fourth paragraph, I suggest you adjust the sentence to the plural and suppress the term “moderate”, in case you don’t have provided any parameter for that qualitative interpretation of the Pearson’s correlation coefficient previously in the manuscript.**

Table 1 reports Pearson’s correlation coefficient (R), p value, and 95% confidence interval (CI) in the supine and standing positions for HR and HRV parameters against VAS.

**Also, please, discuss the significant correlation between VAS and RMSSD observed in standing position.**

As requested, we added the following sentence:

*Most of these correlations were found when HRV was measured in supine position, except for RMSSD in standing position. This later result emphasizes the value of RMSSD as a key parameter of HRV analysis.*

**Limitations:
This section was really improved. Congratulations.**

Thank you! We have added one more limitation on VAS

**Please, change “is” to “was” in the following sentence of the limitations: “The separation criterion between the WB+ and WB- groups is arbitrary”.**

Done **Conclusion
The adjustments made in conclusion were nice. Congratulations.

In my opinion, changing: ”general cardiovascular detraining” to “cardiovascular health” may provide to the reader a more assertive sentence. Feel free to incorporate or not that suggestion.**

We agree and modified as suggested. **Regarding the last sentence of the conclusion, please rethink the term “general population”, especially considering that, although broad, your sample may not represent the general population. In my opinion, simply changing “in the general population” to “in this population” may solve this issue.**

Done. Thank you! **Table 1:
Please report the measurement units of HR and HRV indexes in the first column of table 1.**

Done.

**Figures 1 and 2:

Figure legends describe the following symbols: “* p < 0.05 for difference with
BSL. # p < 0.05 for difference with CFN.”. However, in the figures 1 and 2 the symbols are one or two asterisks “*” or “(**)”. Please, clarify what the two asterisks symbol means.**

We added the missing information.

** p < 0.05, ** p < 0.01 for difference with BSL*
