# Supplementary material for: Effects of COVID-19 lockdown on heart rate variability
Source: PLoS One. 2020 Nov 12;15(11):e0242303. doi: 10.1371/journal.pone.0242303 (PMC7660489; doi:10.1371/journal.pone.0242303)
Supplement: Attachment — Submitted filename: Reviewer comments.docx [file pone.0242303.s002.docx]

**Reviewer #1: In this paper, the authors investigated changes in heart rate variability before, during and after the lockdown which took place for about two months in France.**

We thank you for reviewing our manuscript and for providing comments and suggestions that have helped us to improve it. We have considered your remarks and made amendments when necessary in the revised manuscript. We appreciate your further perusal of the revised manuscript.

We have provided our responses to your comments that are in **bold**. Amended sentences are in italic with the additional wordings in red.

**Data was collected according to standard procedure (morning test), and all typical metrics are ported, in the time (rMSSD) and frequency (LF HF) domains, for a decent sample of 95 individuals

Most importantly, subjective data was collected together with objective physiological data, which I find paramount in this case considering that despite what much of the literature reports, physiology needs to be contextualised with other variables, as exemplified by the fact that HRV could go either way (assuming a similar response for all individuals would be naive), and indeed this change does relate with the objective measurement of physiological stress derived via HRV analysis

The sample gets a bit small when stratified (e.g. only 5 women in the WB+ group), however as the changes are analysed relatively to their own historical data (baseline), I would consider this issue less problematic than in the case of comparing groups**

Thank your for this comment, indeed we designed the analysis so that each individual is his own baseline which minimizes the potential bias when the groups have unbalanced number. **My speculation for the lack of change in the standing position might be linked to the protocol, often the orthostatic measurement creates more issues than it tries to resolve, with individuals getting anxious or too worked up by the quick change of posture, which might result in the test itself just capturing that transitory readjustment, which has little to do with baseline physiological stress - which is what the test aims at capturing. It could be helpful in this case simply to do two separate tests, maybe separated by a few minutes, but this procedure is of course less practical. Unfortunately, in the paper it is not clear if the protocol required a short period between the two phases (e.g. less than 30 seconds) or not, and therefore if this speculation makes sense. Please revise the paper including more information about the exact protocol, and also discuss this aspect if applicable at that point.**

Thank you for this comment. The transition between the supine and standing phases was a few seconds (typically between 3 and 8 seconds). We agree that the standing phase induces a lot more issues than the supine one, but in our view (and before the pandemic crisis broke out) the supine phase is designed to assess the baseline physiological stress while the standing phase is designed to check for lack of activation of the sympathetic system or lack of withdrawal of the parasympathetic system. It is not designed to assess baseline physiological stress. For clarity, we added the following sentences in the Methods section in the manuscript:

The participants were walked through the test by the dedicated mobile application, they started the recording from the application, in the supine position. After five minutes the smartphone beeped, at which point the participants immediately stood and pressed a button on the application to start the recording of the standing phase. After five minutes in the standing phase the smartphone beeped again to inform that the test was over. The transition phase between the supine and the standing positions last 3 to 8 s on average. The HRV analysis does not take into account the first minute of recording in each position.  **Interesting to see also how the change in ANS activity was rather stable during CFN

I understand that this is not the focus of the paper, but it would be relevant for practitioners and users of these technologies if the authors could speculate on what is behind the differences, why a smaller group felt better (which was reflected in their ANS) and why a larger group had more issues, any how this second group could potentially make changes in case another lockdown happens, to favore a more positive response**

Thank you for this suggestion. The factors influencing a positive or negative response to lockdown are numerous, complex, and often interact between each other. In addition, most factors are out of hands of the practitioners. We can only speculate that encouraging physical activity (but still respecting the lockdown rules) even simply walking and reassuring the population with regards to the numerous stressors (financial loss, fear of infection, lo of social activities etc.) may be the most effective ways to counteract the deleterious effects of lockdown.

**Given that the data was collected as part of other studies, I wonder if any other potential correlates were collected (physical activity apparently not), but anything else that could shed some light on factors behind these differences would be relevant)**

Thank you for this excellent suggestion, but unfortunately, we do not have anything substantial that could shed light on the factors explaining the differences between the groups. The strict lockdown made impossible any visit to the laboratory so that no complementary measures were recorded. **In this context I find the discussion section quite limited by the focus on physical activity. In my experience, large changes in heart rate and rMSSD baseline are hardly associated to changes in physical activity alone, especially over rather short time periods and in healthy individuals, while psychological stressors might have a much larger impact. Given that the lockdown is clearly a huge psychological stressor, I would expect more discussion points around this aspect.**

We added the following paragraph in the discussion section:

Beyond this speculative influence of physical activity, there are many stressors during lockdown [2]: subjective (i.e., poor information on the duration of the lockdown, the fears of infection, frustration and boredom, sense of isolation, anger , guilt, helplessness, loneliness, nervousness, sadness) or objective (i.e., inadequate supplies, financial concerns, altered usual day-to-day activities etc.) sources of stress have an impact on psychological outcomes and indirectly on HRV.

**Note also how your own data seems to highlight how the psychological aspect is key (how are you feeling is the question, which does not seem to be tuned to physical activity), more than the physical activity aspect. Of course lack of physical activity as per se a strong psychological effect especially in previously active people, but then the conversation is not about de-training, it's again about psychological wellbeing when unable to perform physical activity. Hopefully this makes sense to the authors and the discussion can be extended to better cover these aspects, which of course are all speculative since no data has been collected on other covariates**

We entirely agree with these suggestions, we have added the following in the discussion section

Through questionnaire in the application and for each test, the participants declared their potential lack of sleep, alcohol consumption (as limited as possible), digestive problems and other potential confounding factors that may have modified the HRV parameters reported in this study. Those factors are known as key in HRV and well-being alterations and may partly explain the cardiovascular deconditioning observed. However, in the present study, we did not find any factor that may have explained the differences between the two groups.

**Reviewer #2: The manuscript presents recordings of heart rate variability (HRV) and subjective well-being in a sample of French healthy adults before, during and after a period of strict confinement, imposed by the progression of the COVID-19 pandemic.**

**Using a subjective well-being metric as an independent variable, the psychophysiological response (measured by HRV indices recorded in different postures) was compared for groups and moments. Association between subjective well-being and HRV were also investigated.
The data recorded by the authors may present valuable and novel information regarding well-being and autonomic modulation of heart rate responses to a critical condition as the strict confinement experienced by the subjects of the sample. However, some analysis lacks justification (a priori) while other methodological issues deserve some attention. Also, the discussion and conclusions sound somewhat speculative or shallow.
In general, the study may present valuable contributions to the field, but some major adjustments are proposed hoping to contribute to the production of an even better manuscript.**

We thank you for reviewing our manuscript and for providing comments and suggestions that have helped us to improve it. We have considered your remarks and made amendments when necessary in the revised manuscript. We appreciate your further perusal of the revised manuscript.

We have provided our responses to your comments that are in **bold**. Amended sentences are in italic with the additional wordings in red.

**Abstract
Considering the suggestions made in the methods and results sections that can significantly change the content of the manuscript body, it may not be worthwhile to address the abstract section in detail now. However, here are some suggestions:
I think that the readability could be improved if the acronyms (“BSL”, “CFN” and “RCV”) were placed next to the related terms. Since it is a personal impression, please, feel free to adopt or not that adjustment.**

Thank you for this suggestion the abstract now reads:

…on a regular basis before *(BSL),* during *(CFN)* and after *(RCV)* the lockdown.

**A brief sample characterization could be appreciable in this section, also a succinct presentation of the analysis made may help the reader to orient himself in relation to the reported results.**

95 participants were included in this study (27 women, 68 men, 37 ± 11 years, 176 ± 8 cm, 71 ± 12 kg),

**The acronyms “WB+” and “WB-” appear in the results without properly previous presentation. Please, provide a brief explanation for what they mean.**

the methods section of the abstract now reads

The participants were split in two groups, those who reported an improved well-being (WB+, increase >2 in VAS score) and those who did not (WB-) during CFN.

**Its not clear in the abstract (as also in the manuscript body) of which moment (BSL, CFN, or RCV) the correlations refer to. Please, clarify that aspect.**

You are right. We modified the abstract as follows:

When pooling data of the three phases, there was a moderate significant correlation

New paragraph was also added in the manuscript body:

## Correlations

Correlations were computed for LF, HF, HR and RMSSD against VAS, in the supine and the standing positions, regardless of the time points (BSL, CFN and RCV), which means that all data points were pooled when computing the correlations. Correlations were made using MATLAB^®^ (R2019a, MathWorks, Natick, MA, USA)

**Regarding the abstract conclusions, I suggest that the authors stick only to what the results allow to conclude. To assume that 20% of the subjects had an improvement in the autonomic status goes somewhat beyond the possibilities that the results of the manuscript allow. The changes in the average values of RMSSD and HR of the two groups in the three periods in which the recordings were made can reflect a number of things, including the return to a normal status, considering an anticipatory stress effect that would already be present in some individuals of the sample, in view of the progression of the COVID-19 disease in the world (which starts in December 2019).**

We entirely agree with this suggestion and we have turned down the tone of the discussion and conclusion accordingly.

**Also, the group division, without previous justification, anchored in a metric, apparently without prior validation, raises some concerns. In addition, the arbitrary determination of a value "> 2" as a cutoff point raises another concern. The conclusion is based mainly on the results that come as consequences of these decisions that were at first not justified, and this greatly weakens the assertiveness and weight of the sentences.**

You are right. We toned down our conclusion.

Our results suggest that the strict COVID-19 lockdown likely had opposite effects on French population as 20% of participants improved parasympathetic activation (RMSSD, HF) and rated positively this period

**At the end of the adjustments resulting from this review, I suggest that the authors rethink the abstract and, if possible, adopt a more cautious language taking into account what can actually be concluded in view of the methodological characteristics and results presented by the study.**

As recommended, we modified the conclusion of the abstract, as follows:

The observed recordings may reflect a large variety of responses (anxiety, anticipatory stress, change on physical activity…) beyond the scope of the present study. However, these results confirmed the usefulness of HRV as a non-invasive means for monitoring well-being and health in the general population

**Introduction
PARAGRAPH 1:
The first part of the first paragraph develops well. When it states: “resulted in decreased physical activity”, I suggest adopting a more measured language. Although we agree that confinement may have resulted in a reduction in physical activity, the reference cited does not supports the statement as it is made. In the next sentence, data from the national institute “Santé Publique France” are presented, I suggest including this source properly in the references and then citing it. At the end of the first paragraph the authors mention that the period of confinement may have caused changes in “cardiovascular fitness and psychological states”, I suggest complementing this sentence with other references such as: Brooks et al., 2020 (https: //pubmed.ncbi.nlm.** [**nih.gov/32112714/**](http://nih.gov/32112714/)**), and Wang et al., 2020 (**[**https://www.ncbi.nlm.nih.gov/pmc/articles/PMC7084952/**](https://www.ncbi.nlm.nih.gov/pmc/articles/PMC7084952/)**); as also mentioning the impact on metabolism and body composition (fat mass and free fat mass) among the possible consequences of confinement, as noted in Martinez-Ferran et al., 2020 (**[**https://pubmed.ncbi.nlm.nih.gov**](https://pubmed.ncbi.nlm.nih.gov/) **/ 32466598 /).**

Thank you for those very relevant suggestions, first paragraph now reads

, may have resulted in decreased physical activity.

[…]

. Beyond these self-declared data, the impact of the lockdown period remains unclear, strong psychological impacts have been reported in a review [2] and specifically to the COVID-19-related lockdown [3], as well as metabolic and body composition outcomes [4], but one may hypothesize that it may have led to changes in cardiovascular fitness and psychological states.

**PARAGRAPH 2:
The first sentence of the second paragraph could be improved if the references cited were complemented, especially with regard to the exercise's ability to mitigate the psychological effects of confinement, since Pareja-Galeano (2015 - https: //pubmed.ncbi.nlm.nih. gov / 25730691 /) addresses in his letter to the editor the interaction between types of exercises and their possible effects specifically on the symptoms of depression in response to a study that investigates the relationship between PA and symptoms of depression in adolescents.**

The beginning of paragraph 2 now reads:

Exercise plays a fundamental role in cardiovascular health [5] but also in mitigating the psychological impacts of lockdown [6]. Aerobic exercises such as walking or endurance running appears to be more beneficial than sprint running or force/power exercises [6,7].

**I also suggest to complement the second sentence of the same paragraph addressing applications of HRV in contexts and populations more related to the theme of the present study as in Thayer et al., 2010 (**[**https://pubmed.ncbi.nlm.nih.gov/19910061/**](https://pubmed.ncbi.nlm.nih.gov/19910061/)**), Hynynen et al., 2011 (**[**https://pubmed.ncbi.nlm.nih.gov/20972879/**](https://pubmed.ncbi.nlm.nih.gov/20972879/)**), Laborde et al., 2017 (**[**https://pubmed.ncbi.nlm.nih.gov/28265249/**](https://pubmed.ncbi.nlm.nih.gov/28265249/)**), and Kim et al., 2018 (**[**https://pubmed.ncbi.nlm.nih.gov/29486547/**](https://pubmed.ncbi.nlm.nih.gov/29486547/)**) in addition to athletes, as in the references already adopted.**

The sentence now reads:

Heart rate variability (HRV) is a commonly used method for cardiovascular follow-up in athletes [8], healthy people [9] cardiovascular patients [10], and is related to self-regulations at the cognitive, emotional, social and health levels [11,12].

**Still on this paragraph, I suggest to rethink some sentences referring to the LF HRV index, in the light of the references suggested above, as well as those that follow: Goldberger 1999 (**[**https://pubmed.ncbi.nlm.nih.gov/10199852/**](https://pubmed.ncbi.nlm.nih.gov/10199852/)**), Medeiros et al., 2018 (**[**https://pubmed.ncbi.nlm.nih.gov/29619594/**](https://pubmed.ncbi.nlm.nih.gov/29619594/)**), and Hayano & Yuda 2019 (**[**https://pubmed.ncbi.nlm.nih.gov/30867063/**](https://pubmed.ncbi.nlm.nih.gov/30867063/)**). Focusing the paragraph on the most reliable indexes and that present better physiological description such as RR, RMSSD and HF may provide a safer way to discuss the results.**

We agree and have modified the paragraph as follows:

Specifically, HRV analysis allows to evaluate the modulation of the sympathetic and parasympathetic branches of the autonomic nervous system. The low-frequency (LF) band reflects a mix of sympathetic and parasympathetic modulations on the heart [13] and has been proposed as an index of the sympathovagal balance [14]. The migration of the respiratory sinus arrhythmia in the low-frequency band is a subject of debate [15], which leaves the physiological implications of this frequency band unclear [16,17]. Respiratory sinus arrhythmia is the main phenomenon provoking changes in the HF band and RMSSD which therefore mainly reflects parasympathetic influences on the heart [18].

**In addition, the part that refer to the use of HRV for performance related ends do not seems to have a direct connection with the theme of the study, please, better elucidate this connection or suppress that part.**

The sentences and references linked to performance have been removed

**Finally, the paragraph ends with a statement that cannot be supported by the cited reference, which presents only associations between HRV (adopted a priori as an indicator of stress) and measures of PA, Cardiorespiratory Fitness, and body composition. It is a cross-sectional study with a sample of men only, which is not a validation study and that does not involve investigating the reliability of HRV. This sentence must be rewritten, and other references can be adopted for this purpose (Thayer et al., 2010** [**https://pubmed.ncbi.nlm.nih.gov/19910061/**](https://pubmed.ncbi.nlm.nih.gov/19910061/)**; Kim et al., 2018 https: // pubmed.** [**ncbi.nlm.nih.gov/29486547/**](http://ncbi.nlm.nih.gov/29486547/)**).**

Finally, HRV-based methods may be used to assess autonomic imbalances, diseases [10], stress and well-being [12].

**PARAGRAPH 3:
Please provide an adequate justification for the present study. Adopt also a more cautious language regarding the objectives since the methods and other characteristics of the study do not allow some of the author's purpose objectives. I’m afraid that there is no evidence for expecting that part of the sample of the investigated population would “appreciate the confinement period”, it is difficult to conceive that this hypothesis could be postulated a priori. Please, provide a rationale for that, otherwise adjust that part.**

We followed your recommendations modified the hypotheses.

We hypothesized that alteration in parasympathetic-related markers (i.e., decreases in RMSSD and HF, increase resting HR) would reflect a negative feeling over the period, whilst a minority of the population would report an improved subjective feeling with consequences on HRV, potentially in line with the report by Santé Publique France [1].

**Method
Please verify the first line of the study design subsection, it’s possible that the word “were” has been suppressed between the words “rules” and “applied”, verify if that sentence is correctly worded.**

Strict lockdown (further denoted CFN) rules were applied in France over 8 weeks, from 17 March to 11 May 2020.

**Experimental design:
I’m afraid that some information regarding the study design are missing in that subsection, while some are placed in other subsections and may better apply to the current. I would suggest the authors to offer a broader and clearer description of the study. Please, in addition to the information regarding the HRV recording periods state the other metrics adopted (VAS) and a concise declaration of the analyses made with those metrics (e.g. that the moments were compared and associations were tested) to better guide readers through the manuscript section.**

For clarity, we added detailed information in the ‘methods’ section

Nevertheless, data collection was performed for the purpose of this study, using recording devices distributed to the participants before the lockdown. The equipment consisted of a RR-interval-measuring chest strap and a dedicated mobile application that recorded both RR intervals from the strap and a declared visual analogic scale (VAS).

The participants were walked through the test by the dedicated mobile application, they started the recording from the application, in the supine position. After five minutes the smartphone beeped, at which point the participants immediately stood and pressed a button on the application to start the recording of the standing phase. After five minutes in the standing phase the smartphone beeped again to inform that the test was over. At that point, the participants were asked to fill-up the VAS by sliding the cursor according to their sensation of the moment. The transition phase between the supine and the standing positions last 3 to 8 s on average. The HRV analysis does not take into account the first minute of recording in each position.

**Participants:
Have the authors a good reason to not include subjects with more than 60 years of age? It would be interesting provide a rationale to that age range inclusion criteria adoption.**

As stated in the manuscript, the lockdown was unexpected, the participants were equipped with the devices for other purposes than this study in the first place. The initially planned studies involved physical exercises with a maximum age for inclusion criteria of 60 years-old to limit the cardiovascular risks. We agree that it is a limitation regarding the effect of COVID-lockdown.

**Also, regarding the inclusion criteria, please explain how the data was collected, clarify which criteria were just declared by the participants and which were really measured.**

Nevertheless, data collection was performed for the purpose of this study, using recording devices distributed to the participants before the lockdown. The equipment consisted of a RR-interval-measuring chest strap and a dedicated mobile application that recorded both RR intervals from the strap and a declared visual analogic scale (VAS).

**Please, justify or clarify why at least forty orthostatic tests were adopted as inclusion criteria during the period of the study, if a total of just thirty where necessary, as you used 10 tests in each period for comparisons.**

From January 1^st^ to May 11^th^ (end of lockdown) there were 20 weeks. At 2 tests per week in average makes 40 tests to have a reasonable follow-up. In addition, if all the tests were performed in one of the three periods (baseline for example) there was no point comparing the periods, hence our additional criteria for at least 10 tests in each period, again to have a reasonable follow-up.

**Heart rate variability:
Since the inter-beat intervals (IBI) were self-recorded, please, state explicitly what instructions were given to participants and if any prior training has been offered for the subjects to properly perform the IBI recordings. Please, include the validation studies regarding the IBI recording devices and for the smartphone app adopted for the IBI recording. Otherwise, mention that in the study limitations.**

The H7 is the most used RR-recording device, the TP5+ uses the exact same technology:

*Comparison of Heart-Rate-Variability Recording With Smartphone Photoplethysmography, Polar H7 Chest Strap, and Electrocardiography. Daniel J Plews, Ben Scott, Marco Altini, Matt Wood, Andrew E Kilding, Paul B Laursen. Int J Sports Physiol Perform. 2017 Nov 1;12(10):1324-1328. doi: 10.1123/ijspp.2016-0668. DOI: 10.1123/ijspp.2016-0668*

The smartphone application is just a Bluetooth receiver, using the standard BLE protocol, which does not interfere in any ways with the recording. It is just a wireless system. The results would have been exactly the same if the RR intervals had been stored on the smartphone memory and downloaded to a computer using a wire. https://web.archive.org/web/20170130085524/https://www.bluetooth.com/specifications/bluetooth-core-specification

**Regarding the artifact corrections, please, declare if any error percentage were adopted as exclusion criteria for IBI recordings and which error percentage it was. Otherwise, provide a rationale for it, since some error percentages may profoundly affect the reliability of the IBI recording and resulting HRV values (Peltola, 2012** [**https://pubmed.ncbi.nlm.nih.gov/22654764/**](https://pubmed.ncbi.nlm.nih.gov/22654764/)**). Please, also declare the recording time window adopted in each position for the HRV indices calculation and, if some stabilization period was adopted, include that information too.**

Our quality check goes beyond just having the number/percentage of artifacts, it uses other measures such as closeness of the artifacts, etc. In any case the quality of the signals was checked to ensure that artifacts did not drastically affect the extracted HRV parameters. Those are in-house algorithms under patenting process; therefore, we cannot disclose in detail how they work.

Out of each of the 5-minute periods, the first minute was used as a stabilization period and was excluded from the analysis whilst the last four minutes were analyzed for HRV.

**Finally, please, state how did you handled with the following aspects that could acutely interfere in the HRV recordings: alcohol consumption, quality and duration of sleep, effects of vigorous exercises and other known factors. If those issues were not properly addressed, please, declare in the study limitations.**

Through questionnaire in the application and for each test, participants declared their potential lack of sleep, alcohol consumption (as limited as possible), digestive problems and other potential confounding factors that may have modified the HRV parameters reported in this study. Those factors are known as key in HRV and well-being alterations and may partly explain the cardiovascular deconditioning observed. However, in the present study, we did not find any factor that may have explained the differences between the two groups.

**Visual analogic scale:
Please, provide the validation study that support the adopted well-being metric. Otherwise, mention that in the study limitations. Also, clarify what means the graduation values 0 to 10 that the VAS referred to, and declare if the subjects received any prior training to use that mobile application.**

VAS is a widely used tool electronically

*Visual analogue scales (VAS): Measuring instruments for the documentation of symptoms and therapy monitoring in cases of allergic rhinitis in everyday health care*

*Position Paper of the German Society of Allergology (AeDA) and the German Society of Allergy and Clinical Immunology (DGAKI), ENT Section, in collaboration with the working group on Clinical Immunology, Allergology and Environmental Medicine of the German Society of Otorhinolaryngology, Head and Neck Surgery (DGHNOKHC). Allergo J Int. 2017; 26(1): 16–24. doi: 10.1007/s40629-016-0006-7*

Becare S.A. employees controlled that the participants properly used the mobile application and chest belt and responded quickly to any question from the participants using dedicated chat bots, emails or telephone.

**Statistical Section
Importantly: Regarding the group effect division adopted here (“WB+” and “WB-”), no rationale or justification was presented a priori for that division. So, the authors must explain in the manuscript why that division was adopted, which criteria were adopted for the group division and justify them, preferably based on the subject literature.**

We adopted the criteria of change in VAS >2, that corresponded to 20% change in the scale. We expected with such a large change to lower the intra-individual variability. As explained in ‘methods’, the analysis is based on a large number of records.

Another important limitation of the present study is that our sample did not allow any other group divisions, evidently justifiable by literature, as by age clusters or gender that may be predictors of negative psychological impacts of lockdown. Moreover, Hnatkova et al. [27] showed that heart rate responses to postural changes may be different for sex.

**I would like to add that some other group divisions, more evidently justifiable by literature, as by age clusters or by sex could be made instead of the adopted by the authors, since Brooks et al., 2020 (cited in the present manuscript references) highlighted that gender and age may be predictors of negative psychological impacts of quarantine and Hnatkova et al., 2019 (**[**https://pubmed.ncbi.nlm.nih.gov/31611089/**](https://pubmed.ncbi.nlm.nih.gov/31611089/)**) showed that heart rate responses to postural changes may be different for sex.**

We would have liked to be able to conduct such studies grouping age, gender and postural changes. But that required to have a priori groups matched for those parameters which obviously we did not have as the lockdown was unexpected. As stated in the limitations section, despite we had no prior intention to study the lockdown and failed to have such evenly constituted groups, we feel like we have a dataset that still shows some interesting results which should be brought to the readers. We added the following sentences in ‘limitations’ section

Another important limitation of the present study is that our sample did not allow any other group divisions, evidently justifiable by literature, as by age clusters or gender that may be predictors of negative psychological impacts of quarantine. Moreover, Hnatkova et al. (2019) showed that heart rate responses to postural changes may be different for sex.

**Please, present the statistical power achieved with that sample for the analysis used.**

Statistical power was added in the result section.

**The authors must declare if unitary or averaged values of the individuals HRV recordings were adopted in the different moments (BSL, CFN and RCV) for the comparisons made in the two-way ANOVA analysis. Also, regarding both metrics (HRV and VAS), clarify which values (unitary or averages) and of which moments (BSL, CFN and RCV) were adopted for the calculation of Pearson’s correlation coefficient. Please, provide information in a more detailed way in this topic, if possible.**

ANOVA analyses were carried out on unitary values (i.e. no average per period or otherwise). Pearson’s correlation coefficients analyses were carried out on unitary values (i.e. no average per period or otherwise).

**Results
If some data is placed in tables, it could improve that section.
Please, present the HR and HRV indices means in each period (BSL, CFN and RCV) per condition (SU and ST). Please, present the correct units in the figures 1 and 2. Also, in figure legends state of which recording positions they refer to.**

Figures 1 and 2 have been updated to also show the standing position. Units of the figures are normalized with respect to baseline as stated in the statistics section.

HR and HRV units were normalized with respect to BSL.

Figures legends have been updated to indicate the position.

**Figure 3 is missing. Regarding the correlations, please clarify the points above mentioned in methods section. If correlations were traced in each one of the moments (BSL, CFN and RCV), please, present all Pearson’s correlation coefficients and respective confidence intervals, if possible.**

Apologies, Figure 3 never existed. Table 1 has been added with all Pearson’s coefficient values.

**I would also suggest a regression analysis combining FC and HRV indices, since there are some interactions between those variables that can highlight or suppress the observable psychophysiological effects of interest (De Geus et al., 2018** [**https://pubmed.ncbi.nlm.nih.gov/30357862/**](https://pubmed.ncbi.nlm.nih.gov/30357862/)**)**

Here are the Pearson’s correlation data of HRV vs. HR data

|  | R | p | CI |
| --- | --- | --- | --- |
| LF SU | -0.22 | <0.01 | -0.35 -0.08 |
| HF SU | -0.31 | <0.01 | -0.49 -0.10 |
| RMSSD SU | -0.52 | < 0.001 | -0.61 -0.41 |
| LF ST | 0.12 | 0.10 | -0.02 0.25 |
| HF ST | 0.03 | 0.70 | -0.11 0.17 |
| RMSSD ST | -0.28 | < 0.001 | -0.40 -0.15 |

We believe that there is no need to show this table in the manuscript. However, if the reviewer asked for its inclusion, we would do it

**Discussion
Most parts of discussion refer to the unjustified group division made by the authors and to a survey relating to behavioral aspects during lockdown as Physical Activity (PA) and exercise, that were not measured or controlled in the present investigation, while the interesting association between VAS and HRV indices was barely discussed. I would like to suggest to the authors a deeper and less speculative discussion, preferably based on the literature pointed out in the introduction and along of the reviewer suggestions.**

We fully agree and acknowledge your relevant suggestions that help to improve the manuscript.

We toned down the influence of physical activity. We deleted the following sentences:

*~~These lifestyles and behaviors included a given level of physical exercise to maintain an adequate health status [23], and guarantee an active aging by reducing the risk associated diseases in older people [24].~~*

*~~In the present study, 80% of the participants reported an increased HR associated to decreased RMSSD and increased LF: these changes could be the consequences of a cardiovascular detraining, with decreased parasympathetic influences on the heart. The loss of outdoor activities as walking is likely of clinical importance since lockdown accelerates vicious circles between isolation, lack of physical activity, psychological stress/anxiety and impaired immunity [27]. Meanwhile, the participants had more time to exercise, but they had to do it by themselves, based on their personal knowledge and material possibilities, with extremely limited external advises and totally absent group motivation.~~*

We also added the following paragraph on the psychological outcomes

Beyond this speculative influence of physical activity, there are many stressors during quarantine [2] : subjective (i.e., poor information on the duration of the quarantine, the fears of infection, frustration and boredom, sense of isolation, anger , guilt, helplessness, loneliness, nervousness, sadness) or objective (i.e., inadequate supplies, financial concerns, altered usual day-to-day activities..) sources of stress have an impact on psychological outcomes and indirectly on HRV.

**Limitations
In addition to the limitations pointed out in other sessions, it would be prudent to comment that factors that may be associated with HRV or modulate their interactions with different stressors have not been controlled, such as: Body composition (Esco et al., 2011 https: //pubmed.ncbi.nlm .**[**nih.gov**](http://nih.gov/) **/ 21691230 /; Yi et al., 2013** [**https://pubmed.ncbi.nlm.nih.gov/23225799/**](https://pubmed.ncbi.nlm.nih.gov/23225799/)**), Cardiorespiratory Fitness (Buchheit & Gindre, 2006 https: //pubmed.ncbi.nlm.** [**nih.gov/16501030/**](http://nih.gov/16501030/)**; Kiviniemi et al., 2017** [**https://pubmed.ncbi.nlm.nih.gov/29135784/**](https://pubmed.ncbi.nlm.nih.gov/29135784/)**) and others.**

We have modified the ‘limitations’ section as follows:

Another important limitation of the present study is that our sample did not allow any other group divisions, evidently justifiable by literature, as by age clusters or gender that may be predictors of negative psychological impacts of quarantine. Moreover, Hnatkova et al. (2019) showed that heart rate responses to postural changes may be different for sex.

Through questionnaire in the application and for each test, participants declared their potential lack of sleep, alcohol consumption (as limited as possible), digestive problems and other potential confounding factors that may have modified the HRV parameters reported in this study. Those factors are known as key in HRV and well-being alterations and may partly explain the cardiovascular deconditioning observed. However, in the present study, we did not find any factor that may have explained the differences between the two groups.

**Conclusion
Some parts of the conclusion sound speculative or walk beyond the possibilities of the study results. Please, considering the reviewer suggestions in the sections above, rethink that section too.**

We modified the conclusion as follows:

The lockdown in France was sudden and severe, inducing an increase in many subjective and objective stressors as well as drastically limiting the possibilities for physical activities. This likely resulted in a stressful situation that may lead to negative psychological outcomes and/or a general cardiovascular detraining (i.e., decreased RMSSD and HF and increased resting heart rate in supine position) in most of the population **References
Please adjust reference number two accordingly.**

Reference adjusted (now reference #1)

**Further to the email sent to you from the academic editor, please accept our sincere apologies but request number 4 was incomplete. It should have read;

Thank you for including your competing interests statement; "I have read the journal's policy and the authors of this manuscript have the following competing interests: Nicolas Bourdillon and Sasan Yazdani are employees of becare S.A."

We note that one or more of the authors are employed by a commercial company: becare S.A.
1.      Please provide an amended Funding Statement declaring this commercial affiliation, as well as a statement regarding the Role of Funders in your study. If the funding organization did not play a role in the study design, data collection and analysis, decision to publish, or preparation of the manuscript and only provided financial support in the form of authors' salaries and/or research materials, please review your statements relating to the author contributions, and ensure you have specifically and accurately indicated the role(s) that these authors had in your study. You can update author roles in the Author Contributions section of the online submission form.

Please also include the following statement within your amended Funding Statement.
“The funder provided support in the form of salaries for authors [insert relevant initials], but did not have any additional role in the study design, data collection and analysis, decision to publish, or preparation of the manuscript. The specific roles of these authors are articulated in the ‘author contributions’ section.”
If your commercial affiliation did play a role in your study, please state and explain this role within your updated Funding Statement.

2. Please also provide an updated Competing Interests Statement declaring this commercial affiliation along with any other relevant declarations relating to employment, consultancy, patents, products in development, or marketed products, etc. 

Within your Competing Interests Statement, please confirm that this commercial affiliation does not alter your adherence to all PLOS ONE policies on sharing data and materials by including the following statement: "This does not alter our adherence to  PLOS ONE policies on sharing data and materials.” (as detailed online in our guide for authors** [**http://journals.plos.org/plosone/s/competing-interests**](http://journals.plos.org/plosone/s/competing-interests)**) . If this adherence statement is not accurate and  there are restrictions on sharing of data and/or materials, please state these. Please note that we cannot proceed with consideration of your article until this information has been declared.

Please include both an updated Funding Statement and Competing Interests Statement in your cover letter. We will change the online submission form on your behalf.

Please know it is PLOS ONE policy for corresponding authors to declare, on behalf of all authors, all potential competing interests for the purposes of transparency. PLOS defines a competing interest as anything that interferes with, or could reasonably be perceived as interfering with, the full and objective presentation, peer review, editorial decision-making, or publication of research or non-research articles submitted to one of the journals. Competing interests can be financial or non-financial, professional, or personal. Competing interests can arise in relationship to an organization or another person. Please follow this link to our website for more details on competing interests:** [**http://journals.plos.org/plosone/s/competing-interests**](http://journals.plos.org/plosone/s/competing-interests) **We apologise for any confusionn this has caused and look forward to receiving your revised manuscript,**
